# *Premna bhamoensis* (Lamiaceae, Premnoideae), a new species from Kachin State, northeastern Myanmar

**DOI:** 10.3897/phytokeys.83.12869

**Published:** 2017-07-20

**Authors:** Yunhong Tan, Derong Li, Yongjun Chen, Bo Li

**Affiliations:** 1 Southeast Asia Biodiversity Research Institute, Chinese Academy of Sciences, Yezin, Nay Pyi Taw 05282, Myanmar; 2 Center for Integrative Conservation, Xishuangbanna Tropical Botanical Garden, Chinese Academy of Sciences, Mengla, Yunnan 666303, China; 3 College of Agronomy, Jiangxi Agricultural University, Nanchang, Jiangxi 330045, China

**Keywords:** China, morphology, Myanmar, *Premna menglaensis*, Xishuangbanna Tropical Botanical Garden

## Abstract

In the present study, we describe and illustrate a new species, *Premna bhamoensis* Y. T. Tan & B. Li (Lamiaceae), from Myanmar. In the 1980s, this species was transplanted from Bhamo County in northeastern Myanmar to the Xishuangbanna Tropical Botanical Garden, Chinese Academy of Sciences. The species shows striking morphological similarity to *P. menglaensis* B. Li, and thus, has been misidentified as the latter for a long period of time. However, morphological comparison revealed that *P. bhamoensis* is distinct from *P. menglaensis* in many aspects. Moreover, literature survey and specimen examinations also indicated that *P. bhamoensis* is undoubtedly different from all seven known congenetic species recorded from Kachin State, Myanmar, and a key for their identification has been provided in this paper.

## Introduction

The genus *Premna* L. is one of the largest woody genera belonging to the mint family, consisting of approximately 200 species distributed mainly in the Old World tropics and subtropics (Verdcourt 1992, Harley et al. 2004). The genus was first described by Linnaeus (1771), on the basis of two species, *P. serratifolia* L. and *P. integrifolia* L., which are now treated as a single species (de Kok 2013). It was traditionally placed in the subfamily Viticoideae Briq. (Briquet 1897, Chen and Gilbert 1994, Harley et al. 2004), but was recently transferred to the newly established subfamily Premnoideae B. Li, R.G. Olmstead & P.D. Cantino (Li et al. 2016).

**Figure 1.**
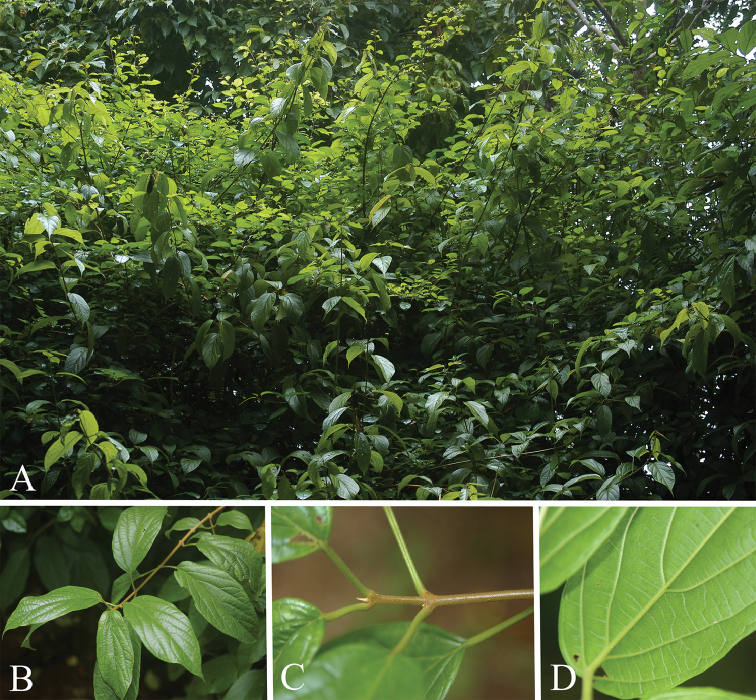
*Premna bhamoensis* Y. T. Tan & B. Li, sp. nov. **A** habit **B** a branchlet with ovate-oblong to elliptic leaves **C** branchlet and petioles covered by dense brownish pubescences **D** abaxial surface of leaf blade.

**Figure 2.**
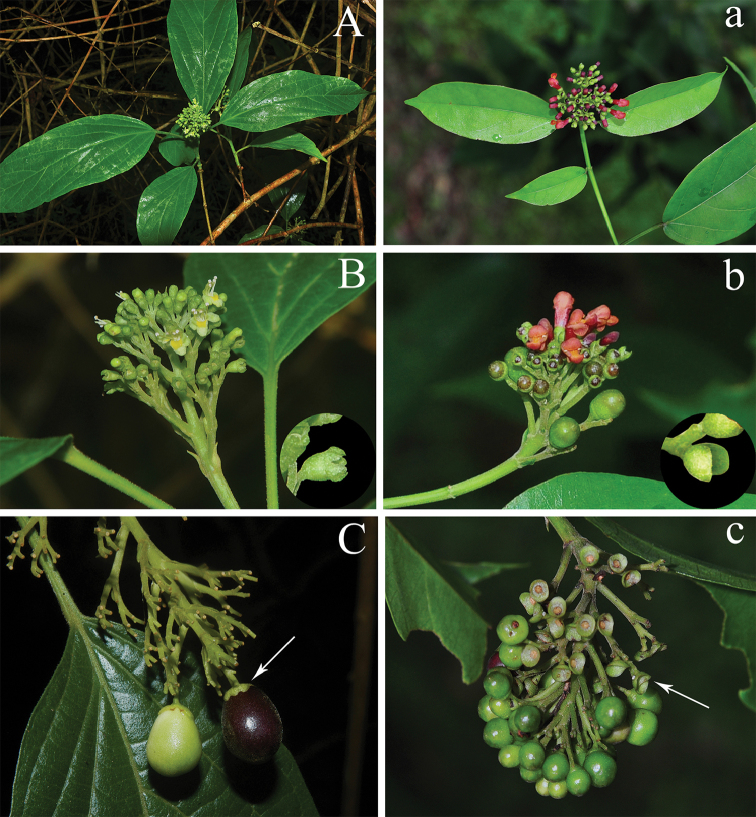
Morphological comparison between *Premna bhamoensis* (**A–C**) and *P. menglaensis* (**a–c**). **A, a** branchlets with inflorescences **B, b** inflorescences, flowers and calyces (in the blank circle) **C, c** fruitescences and fruits (arrow show fruiting calyx).

With 46 species recognized in China, *Premna* is the fifth largest genus in Lamiaceae flora of China (Chen and Gilbert 1994, Li and Hedge 1994). In June 2011, a field survey was carried out to investigate the biodiversity of *Premna* in Yunnan Province, southwestern China. When visiting the Xishuangbanna Tropical Botanical Garden (XTBG), Chinese Academy of Sciences (CAS), the authors found a rare *Premna* shrub in the fruiting stage (Figure 1), which was being cultivated in the C20 region of the garden, and was labeled as “*Premna laevigata* C. Y. Wu” (≡ *P. menglaensis* B. Li, after Li et al. 2013). Superficially, the plant strongly resembles *P. menglaensis* in having a climbing habit, ovate-oblong to elliptic leaves, and a congested pyramid-shaped thyrse (Figure 2), but differs noticeably in having densely pubescent branchlets and petioles, and lips of fruiting calyces distinctly 2- or 3-lobed. Many more differences between this plant and *P. menglaensis* were discovered during the flowering stage, whose observations were taken during the month of May 2012. This analysis indicated that the plant probably represented a new species. In order to verify the information about the origin of this putative new species, we examined the XTBG introduction records and found that this plant was introduced from Bhamo County, Kachin State of northeastern Myanmar in the 1980s. However, precise location data and the collection date had not been recorded. During the period from 2011 to 2016, the first author has visited Kachin State many times, but the plant was not found in this area. Further examination of literature and specimens revealed that seven *Premna* species have been recorded from Kachin State, viz., *P. barbata* Wall. ex Schauer, *P. bengalensis* C.B. Clarke, *P. khasiana* C.B. Clarke, *P. pinguis* C.B. Clarke, *P. pyramidata* Wall. ex Schauer, *P. racemosa* Wall. ex Schauer, and *P. scandens* Roxb. (Kress et al. 2003). However, none of them is morphologically similar to the putative new species. Therefore, it is confirmed that this species of *Premna* is new to science, and thus, we describe and illustrate it in this study.

## Methods

Morphological observations of the new species were carried out based on living plants as well as dry specimens, during the period from 2011 to 2016. Measurements were made using a ruler and a micrometer. Both herbarium and fresh specimens of *P. menglaensis* were examined under a stereo dissecting microscope (StereoZoom® Leica S8 APO, © Leica Microsystems 2017). The conservation status of the new species was evaluated based on the guidelines of the International Union for Conservation of Nature (IUCN 2012). A distribution map was prepared using data obtained from our field observations, herbarium specimens and relevant literature (extrapolated or approximated with respect to a few old or vaguely specified localities).

## Taxonomy

### Premna bhamoensis

Taxon classificationPlantaeLamialesLamiaceae

Y.T. Tan & B. Li sp. nov.

urn:lsid:ipni.org:names:77164217-1

**Figure 3.**
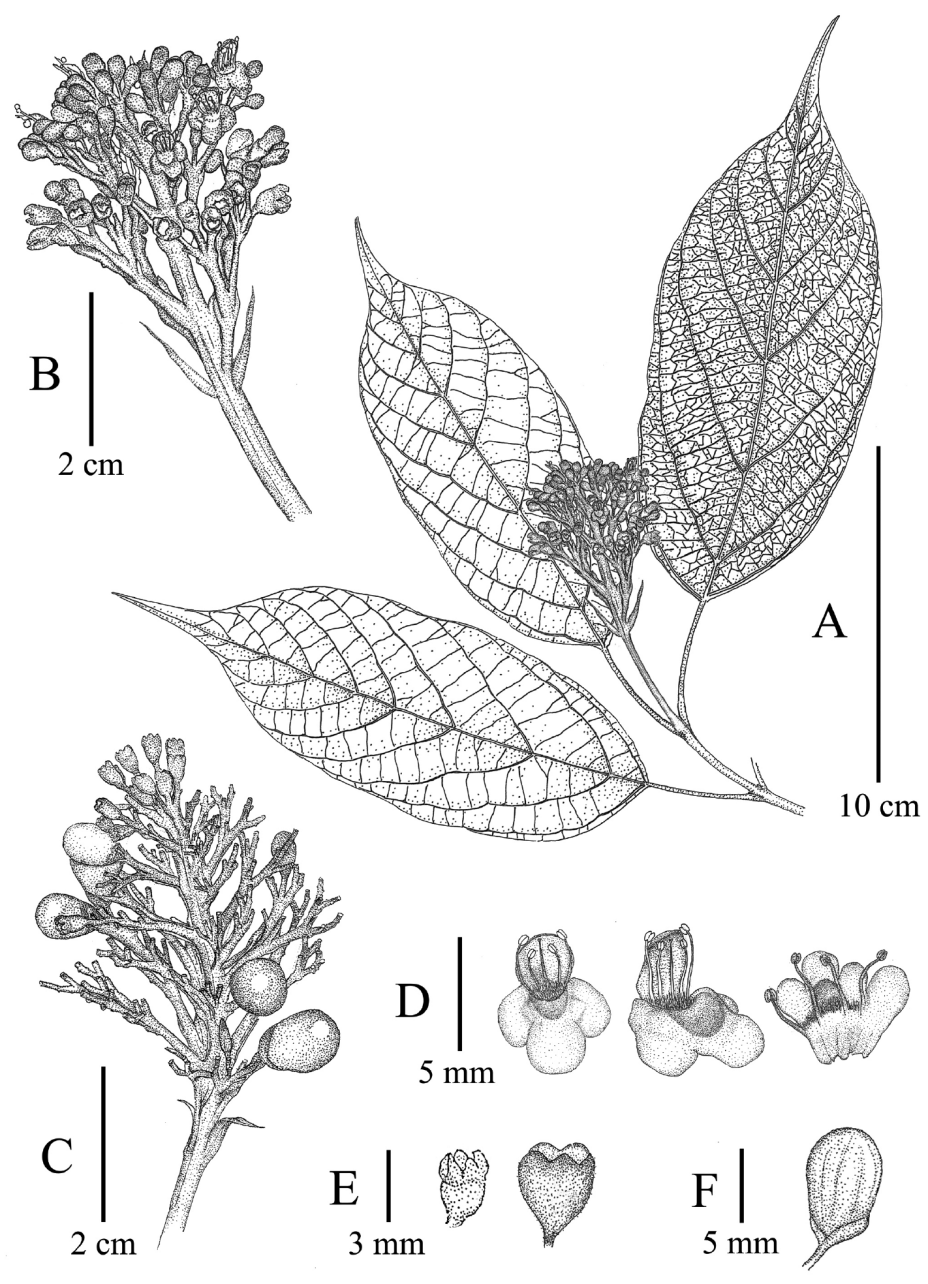
Line drawing of *Premna bhamoensis* Y. T. Tan & B. Li, sp. nov. **A** abranchlets with inflorescence **B** inflorescence **C** fruitescence **D** corolla **E** calyx in flowering (left) and fruiting (right) **F** fruit.

Figures 1 , 2A–C , 3

#### Diagnosis.

The species is most similar in morphology to *P. menglaensis* B. Li, but differs from the latter in having branchlets and petioles densely tomentose (vs. glabrous or glabrescent), leaf blades papery with minute pubescence (vs. leathery and glabrous), flowers green to greenish yellow (vs. red flowers), calyces slightly 2-lipped with five equal lobes (vs. calyces distinctly 2-lipped with entire or minute emarginate lips), and stamens exserted from corolla (vs. included).

#### Type.

MYANMAR. Kachin State, Bhamo County, voucher from a cultivated plant at the Xishuangbanna Tropical Botanical Garden, Menglun Town, Mengla County, Yunnan Province, Alt. 550 m, 21.404408N, 101.152401E, 10 June 2011, *B. Li LB0399* (fruiting branches) (holotype: IBSC!; isotypes: IBSC!, JXAU!, HITBC!).

#### Description.

*Woody shrubs*, climbing. *Branches* brown, terete, with an interpetiolar ridge and sparse small yellow elliptic lenticels, sparsely and minutely pubescent to glabrescent. *Branchlets* grayish to brownish, densely tomentose, without bracts at the base. *Leaves* simple, opposite-decussate, ovate-oblong to elliptic, papery, 9.0–17 × 4.5–7.5 cm, apex long caudate to caudate-acuminate, base cuneate, subrounded to slightly cordate, margin entire; adaxial surface subglabrous except minutely hirsute on veins; abaxial surface densely pubescent with sparse, yellowish-brown glands; veins 4–8 pairs, abaxially raised and adaxially slightly compressed, secondary veins curved and jointed near margin; petiole 1.5–4.5 cm long, furrowed on upper part, densely yellowish-brown pubescent. *Inflorescences* terminal, mostly pyramid-shaped thyrse, densely dusty brownish-yellow pubescent, 4.0–7.5 cm long; peduncles 1.5–2.5 cm long; bracts ovate-lanceolate to lanceolate-linear, 0.6–1.2 cm long, easily deciduous; bracteoles linear or lanceolate-linear, 1.0–2.5 mm long; pedicels 0.5–1.5 mm long. *Calyx* campanulate, 2.5–3.0 mm long, slightly 2-lipped with five equal lobes, apex acute, outside minutely brownish pubescent with brown glands; fruiting calyx distinctly 2-lipped with one lip 2-lobed and another 3-lobed, apex obtuse to subrounded. *Corolla* green to greenish yellow, 2-lipped, 4.5–5.5 mm long; tube 2.5–3.0 mm long, outside glabrous, inside densely white villose around throat; upper lip 1-lobed, entire, broadly oblong-obovate, obovate, concave, apex subrounded, outside glabrous or slightly pubescent; lower lip 3-lobed, middle lobe rounded to obovate, lateral lobes broadly oblong-ovate or ovate. *Stamens* 4, didynamous, filaments greenish-white, glabrous, slightly exserted; anther white. *Ovary* obovoid, 1.0–1.5 mm long, minutely pubescent and golden glandular near the apex; style white, slender, 3.5–4.5 mm long. *Fruits* drupaceous, purplish dark brown, obovoid to obovoid-ellipsoid, 7.0–8.0 × 4.5–5.5 mm, sparsely dusty pubescent and glandular.

#### Phenology.

Flower buds were observed in early April. Flowering was observedfrom mid-May to early June and fruiting from late May to late June.

#### Distribution.

**Figure 4.**
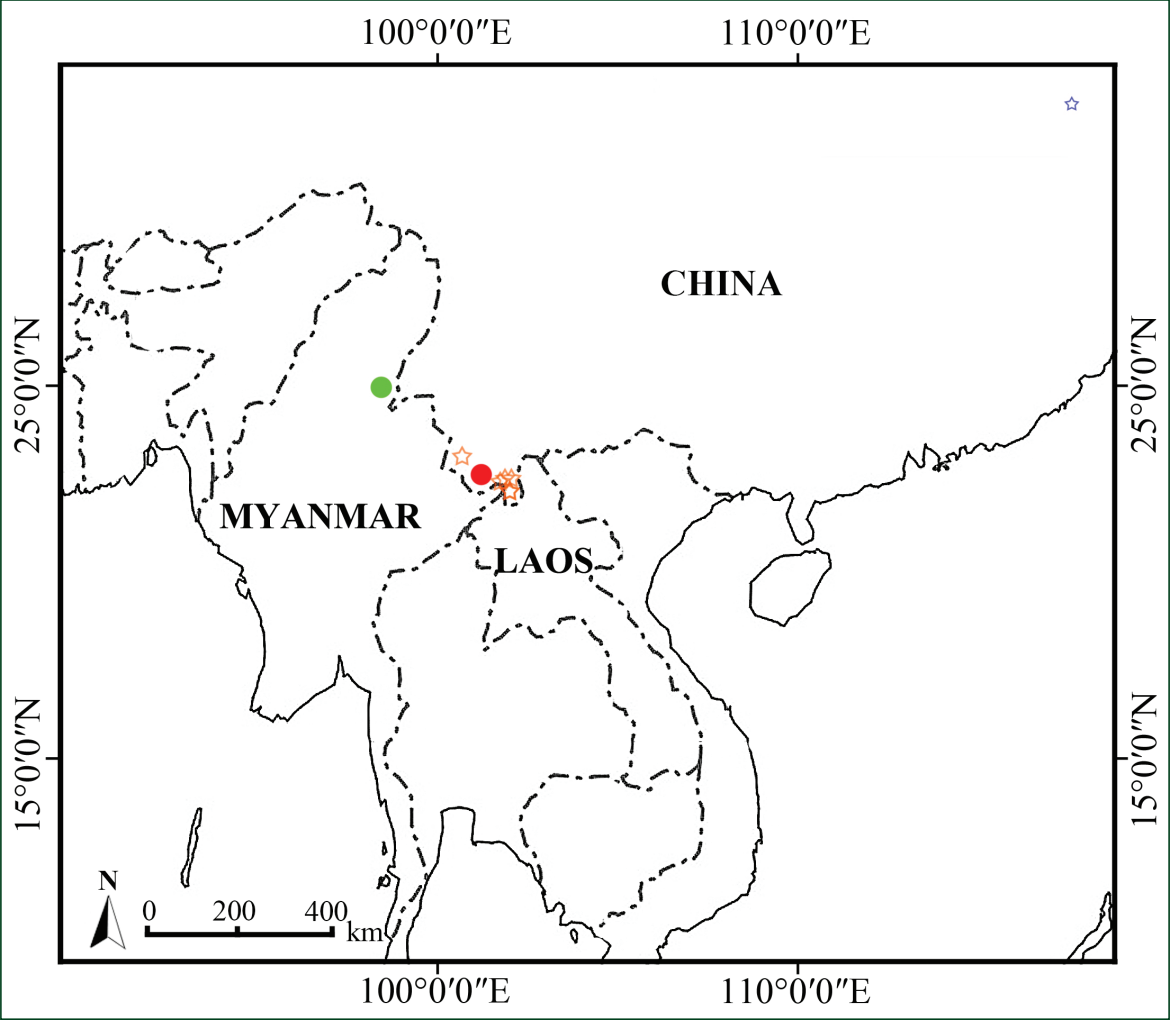
Distribution map of *Premna bhamoensis* (green circle showing the type locality and red circle for the cultivated cite) and *P. menglaensis* (orange star).

Per the introduction record, *P. bhamoensis* is originally collected from northeastern Myanmar, but currently known only from the cultivated type in the Xishuangbanna Tropical Botanical Garden (Figure 4). Based on our experience in examination of Asian *Premna* specimens, we suspect that the species is probably endemic to Kachin State of Myanmar and distributed in a very small area.

#### Etymology.

The specific epithet of this new species, “*bhamoensis*”, is derived from the name of the locality, Bhamo County, from where the species was originally collected.

#### Preliminary conservation status.

Since we have neither rediscovered the wild population of *P. bhamoensis* in Myanmar, nor identified any other specimens in the herbarium, very few details about its natural distribution and/or population status are currently known. Therefore, the information is inadequate to assess the species’ risk of extinction, whether directly or indirectly. In accordance with the IUCN Red List Categories (IUCN 2012), we propose to temporarily list the species as a taxon under the Data Deficient (DD) category. Further field surveys in northeastern Myanmar are needed to gain more information on its abundance and/or distribution.

#### Other specimen examined.

MYANMAR. Kachin State, Bhamo County, voucher from a cultivated plant at the Xishuangbanna Tropical Botanical Garden, Menglun Town, Mengla County, Yunnan Province, Alt. 550 m, 21.404408N, 101.152401E, 31 May 2012, *Y. H. Tan 120* [flowering branches] (XTBG!).

#### Note.

Among the seven *Premna* species recorded in the flora of Kachin State, Myanmar (Kress et al. 2003), *P. pyramidata* (= *P. tomentosa* Willd.) and *P. bengalensis* have dense stellate hairs on branchlets, leaves, and petioles, *P. racemosa* (= *P. interrupta* Wall. ex Schauer) has spikelike inflorescences, and *P. scandens* is a large and glabrous vine. Thus, these four species are quite distinct from *P. bhamoensis*. *P. barbata* and *P. khasiana* both have bracts at the base of branchlets, whereas such bracts are absent in *P. bhamoensis*. *P. pinguis* differs from *P. bhamoensis* in having ovate leaves with strongly serrulate margins, and branches without interpetiolar ridges. All these differences make *P. bhamoensis* a distinct *Premna* species in Myanmar. A key to the *Premna* species in Kachin State of Myanmar is provided below.

Among the Asian *Premna*, *P. menglaensis*, as the introduction label indicated, is the species showing the maximum level of similarity to *P. bhamoensis*. Both are climbing Woody shrubs with ovate-oblong to elliptic leaves, and congested pyramid-shaped inflorescences. However, *P. bhamoensis* can be easily distinguished from *P. menglaensis* on the basis of the differences observed in a number of traits, e.g., branchlets and petioles (densely pubescent vs. glabrous or glabrescent), leaf blades (papery and minutely pubescent vs. leathery and glabrous), flower color (green to greenish yellow vs. red), calyx shape (slightly 2-lipped with five equal lobes vs. distinctly 2-lipped with entire or minute emarginate lips), stamens length (exserted from corolla vs. included) (Figure 2). *P. bhamoensis* also resembles *P. fulva* Craib in having a climbing habit, dense indumentum on branchlets and petioles, green to greenish yellow flowers, and calyces with five lobes, but clearly differs in leaf shape (ovate-oblong to elliptic with entire margins vs. ovate to subrounded with serrate margins) and inflorescence type (congested pyramid-shaped thyrse vs. flat-topped corymbose cyme) (Chen and Gilbert 1994, Tan and Li 2016). Besides, branchlets, petioles, leaf blades, and inflorescences of *P. fulva* are densely covered with long, spreading, golden-brown hairs, which are different from the hairs found on *P. bhamoensis*.

### A key to the species of *Premna* in Kachin State of Myanmar

**Table:** 

1	Inflorescences spikelike	***P. interrupta***
–	Inflorescences compound cymes	**2**
2	Branchlets, leaves, and petioles covering dense stellate hairs	**3**
–	Branchlets, leaves, and petioles glabrous or covering other type of hairs	**4**
3	Leaf blades ovate to ovate-oblong; cymes in a lax conical panicle	***P. tomentosa***
–	Leaf blades elliptic to oblong-lanceolate; cymes in a lax flat-topped corymbs	***P. bengalensis***
4	Vines; branches and leaves glabrous	***P. scandens***
–	Trees, erect or climbing Woody shrubs; branches and leaves pubescent	**5**
5	Base of branchlets surrounded by bracts	**6**
–	Base of branchlets without bracts	**7**
6	Corymbs ca. 15 cm in diameter, peduncles slender; leaf blades turn brownish black when dry	***P. khasiana***
–	Corymbs ca. 4 cm in diameter, peduncles robust; leaf blades brownish yellow when dry	***P. barbata***
7	Leaves margins strongly serrulate; branches without interpetiolar ridges	***P. pinguis***
–	Leavesmargins entirely; branches with interpetiolar ridges	***P. bhamoensis***

## Supplementary Material

XML Treatment for Premna bhamoensis

## References

[B1] Briquet J (1897) Verbenaceae. In: Engler A Prantl K (Eds) Die Natürlichen Pflanzenfamilien, Teil 4, Abt. 3a. Engelmann, Leipzig, 132–182.

[B2] Chen SL Gilbert MG (1994) *Premna*. In: Wu CY Raven PH (Eds) Flora of China, Vol. 17. Science Press, Beijing & Missouri Botanical Garden Press, St. Louis, 16–27.

[B3] Harley RM Atkins S Budantsey AL Cantino PD Conn BJ Grayer R Harley MM de Kok RPJ Krestovskaja T Morales R Paton AJ Ryding O Upson T (2004) Labiatae. In: Kubitzki K Kadereit JW (Eds) Families and genera of vascular plants. Flowering plants. Dicotyledons – Lamiales (except Acanthaceae including Avicenniaceae), Vol. 7. Springer, Berlin, 167–275. https://doi.org/10.1007/978-3-642-18617-2_11

[B4] IUCN (2012) IUCN Red List Categories and Criteria, Version 3.1 (2nd edn). Gland and Cambridge, 32 pp.

[B5] de Kok RPJ (2013) The genus *Premna* L. (Lamiaceae) in the *Flora Malesiana* area. Kew Bulletin 68: 55–84. https://doi.org/10.1007/s12225-013-9433-5

[B6] Kress WJ DeFilipps RA Farr E Yin Yin Kyi D (2003) A checklist of the trees, Woody shrubs, herbs, and climbers of Myanmar (revised from the original works by JH Lace, R. Rodger, HG Hundley and U Chit Ko Ko on the “List of trees, Woody shrubs, herbs and principal climbers etc. recorded from Burma”). Contributions from the United States National Herbarium 45: 1–590.

[B7] Li B Tan Y Zhang ZY Zhang DX (2013) *Premna menglaensis*, a new name for *Premna laevigata* C. Y. Wu (Lamiaceae). Phytotaxa 153: 58–59. https://doi.org/10.11646/phytotaxa.153.1.4

[B8] Li B Cantino PD Olmstead RG Bramley GLC Xiang CL Ma ZH Tan YH Zhang DX (2016) A large-scale chloroplast phylogeny of the Lamiaceae sheds new light on its subfamilial classification. Scientific Reports 6: 34343. https://doi.org/10.1038/srep34343 10.1038/srep34343 PMC5066227 27748362

[B9] Li HW Hedge IC (1994) Lamiaceae. In: Wu CY Raven PH (Eds) Flora of China, Vol. 17. Science Press, Beijing & Missouri Botanical Garden Press, St. Louis, 269–291.

[B10] Linnaeus C (1771) Mantissa Plantarum. Salvius, Stockholm, 587 pp.

[B11] Tan Y Li B (2014) Taxonomic studies on the genus Premna (Lamiaceae) in China—I: the identities of *P. fulva* and *P. tapintzeana*. Phytotaxa 173: 207–216. https://doi.org/10.11646/phytotaxa.173.3.3

[B12] Verdcourt B (1992) Verbenaceae. In: Polhill RM (Ed.) Flora of tropical east Africa. Balkema, Rotterdam, 1–156.

