# Corrigenda: *Premna bhamoensis* (Lamiaceae, Premnoideae), a new species from Kachin State, northeastern Myanmar

**DOI:** 10.3897/phytokeys.85.19983

**Published:** 2017-08-31

**Authors:** Yunhong Tan, Derong Li, Yongjun Chen, Bo Li

**Affiliations:** 1 Southeast Asia Biodiversity Research Institute, Chinese Academy of Sciences, Yezin, Nay Pyi Taw 05282, Myanmar; 2 Center for Integrative Conservation, Xishuangbanna Tropical Botanical Garden, Chinese Academy of Sciences, Mengla, Yunnan 666303, China; 3 College of Agronomy, Jiangxi Agricultural University, Nanchang, Jiangxi 330045, China

A new *Premna* species, named as *Premna bhamoensis* Y.T. Tan & B. Li, was recently described in Tan et al. (2017), however an overlooked spelling error emerged in the authorship of this new species. The name abbreviation of the first author, Yunhong Tan, should be Y. H. Tan, not as originally published Y.T. Tan. Here we correct the authorship of *Premna bhamoensis* as Y.H. Tan & B. Li.
